# Supplementary material for: Spatial transcriptomics maps distinct signatures of human intermuscular adipose expansion in mice
Source: medRxiv. 2026 Jun 26:2026.04.16.26351017. Originally published 2026 Apr 17. Preprint. [Version 2] doi: 10.64898/2026.04.16.26351017 (PMC13127531; doi:10.64898/2026.04.16.26351017)

medRxiv preprint doi: https://doi.org/10.64898/2026.04.16.26351017; this version posted June 26, 2026. The copyright holder has placed this preprint (which was not certified by peer review) in the Public Domain. It is no longer restricted by copyright. Anyone can legally share, reuse, remix, or adapt this material for any purpose without crediting the original authors.

Figure S1

A) Volcano plots for all detected genes comparing expression between IMAT vs muscle (upper panel) and IMAT vs scFAT (lower panel). Genes selected as IMAT specific are highlighted in orange.

B) Sankey plot of top adipocyte-related canonical signaling pathways identified by IPA and enriched in human IMAT-specific genes shown in the Fig. 1A heatmap, with corresponding gene-pathway associations

C) Barplot showing enrichment -log10 p-values for selected adipocyte-related canonical signaling pathways.

D) Representative histological sections of muscle quadriceps in high-fat-diet (HFD) and low-fat-diet (LFD) female mice. Intermuscular adipocytes are indicated by arrows. Scale 100um.

E) Quantitative digital imaging analysis of histological sections in female mice (n = 7-8) after 84 days of HFD and LFD feeding. Data are presented as mean ± SEM.

medRxiv preprint doi: https://doi.org/10.64898/2026.04.16.26351017; this version posted June 26, 2026. The copyright holder has placed this preprint (which was not certified by peer review) in the Public Domain. It is no longer restricted by copyright. Anyone can legally share, reuse, remix, or adapt this material for any purpose without crediting the original authors.

Figure S2

A) UMAP of the murine snRNA-seq reference dataset, colored by cell-type annotation.

B) Dot plot displaying expression levels of the top three marker genes, selected based on mean AUC, for each reference cell type across all identified cell types.

C) Spatial pie chart plot showing spotwise cell-type distributions in LFD and HFD fed male C57BL/6 mice, shown on Visium 10x Genomics slides (n = 2).

D) Boxplots showing the distribution of inferred cell-type proportions in spatial transcriptomics spots from LFD and HFD fed male mice on Visium 10x Genomics slides (n = 2).

E) Bar plot of correlation coefficients comparing spotwise perilipin intensity with cell type content for LFD and HFD fed male mice, acquired on Visium 10x Genomics slides (n = 2).

medRxiv preprint doi: https://doi.org/10.64898/2026.04.16.26351017; this version posted June 26, 2026. The copyright holder has placed this preprint (which was not certified by peer review) in the Public Domain. It is no longer restricted by copyright. Anyone can legally share, reuse, remix, or adapt this material for any purpose without crediting the original authors.

Figure S3

A) Proportional cell type distributions over all 9 identified clusters averaged over all spots and samples (top) and diet specific (bottom). Stacked barplots refer to the mean cell type proportions over all spots for each cluster.

B) Barplot of total spots mapped to the 9 clusters between high-fat diet (HFD) and low-fat diet (LFD) male mice.

C) Dot plot of adipocyte and FAP marker genes from the reference dataset across all nine spatial clusters.

D) Dot plot displaying expression of the top three marker genes for each reference cell type across all nine clusters.

E) Selected Ingenuity Pathway Analysis (IPA) results for genes differentially regulated in HFD compared to LFD male mice, identified across spatial transcriptomic clusters 4, 6, and 7. Color intensity reflects enrichment magnitude (p-value), and pathways are grouped by biological theme to highlight the functional landscape driven by HFD-associated gene expression. Overlaid dots indicate the IPA activation z-score where calculable — pathways lacking a dot either contained fewer than four analysis-ready molecules from the input dataset or showed insufficient directional concordance with curated pathway relationships, precluding z-score estimation.

Figure S4

A) Spearman rank correlation of human IMAT gene signature comparing spot wise expression and cell type proportions in murine skeletal muscle from low-fat diet (LFD) and high-fat diet (HFD) fed male C57BL/6 mice (n = 2).

B) Scatterplot of directed p-values (p * sign (rho)) comparing IMAT specific correlation between spot wise human IMAT gene expression and cell type proportion for all identifued

medRxiv preprint doi: https://doi.org/10.64898/2026.04.16.26351017; this version posted June 26, 2026. The copyright holder has placed this preprint (which was not certified by peer review) in the Public Domain. It is no longer restricted by copyright. Anyone can legally share, reuse, remix, or adapt this material for any purpose without crediting the original authors.

cell types (except adipocytes and FAPs) in LFD fed (x-axis) and HFD (y-axis) fed male mice. Purple dots denote genes significantly correlated in both directions; orange dots denote genes correlated in x-dimension (LFD) only and red in y-dimension (HFD) only. C) Heatmap of human IMAT gene signature in female and male lean, obese and type 2 diabetes (T2D) participants.

medRxiv preprint doi: https://doi.org/10.64898/2026.04.16.26351017; this version posted June 26, 2026. The copyright holder has placed this preprint (which was not certified by peer review) in the Public Domain. It is no longer restricted by copyright. Anyone can legally share, reuse, remix, or adapt this material for any purpose without crediting the original authors.

Figure S5

A) Spatial transcriptomic analysis of genes co-localizing with Ebf2 in C56/Bl6 male mice. Each point represents a gene co-expressed with Ebf2. The x- and y-axes show Spearman correlation coefficients between Ebf2 and the indicated gene in low-fat diet (LFD) and high-fat diet (HFD) barcodes, respectively, calculated using pairwise complete observations. The dashed diagonal indicates unchanged correlation across diets; points above or below the diagonal represent genes with increased or decreased correlation under HFD. Selected genes with the strongest diet-dependent changes are labeled. Ebf2 regulated genes Fabp4 and Cd36 are highlighted in green.

B) Dot plot showing expression of STRING-identified EBF2 interaction genes across nine spatial transcriptomic clusters from LFD and HFD fed male C57BL/6 mice (n = 2).

C) Human skeletal muscle cell (HSkMC) adipogenic differentiation (Diff.) and non-differentiation (Undiff.) comparative analysis by mid-infrared optoacoustic microscopy (MiROM). OA micrograph of lentiviral GFP gene overexpression in HSkMC (upper panel) and parental (wt) HSkMC (lower panel), Big FOV (4 mm × 4 mm) is on the left side, and small FOV (500 μm×500 μm) is on the right side in two channels: red (at 2856 cm^{-1}; CH_2 band - symmetric CH_2 stretching) and cyan (at 1550 cm^{-1}; Amide II band – N-H bending/ C-N stretching).

Figure S1

medRxiv preprint doi: https://doi.org/10.64898/2026.04.16.26351017; this version posted June 26, 2026. The copyright holder has placed this preprint (which was not certified by peer review) in the Public Domain. It is no longer restricted by copyright. Anyone can legally share, reuse, remix, or adapt this material for any purpose without crediting the original authors.

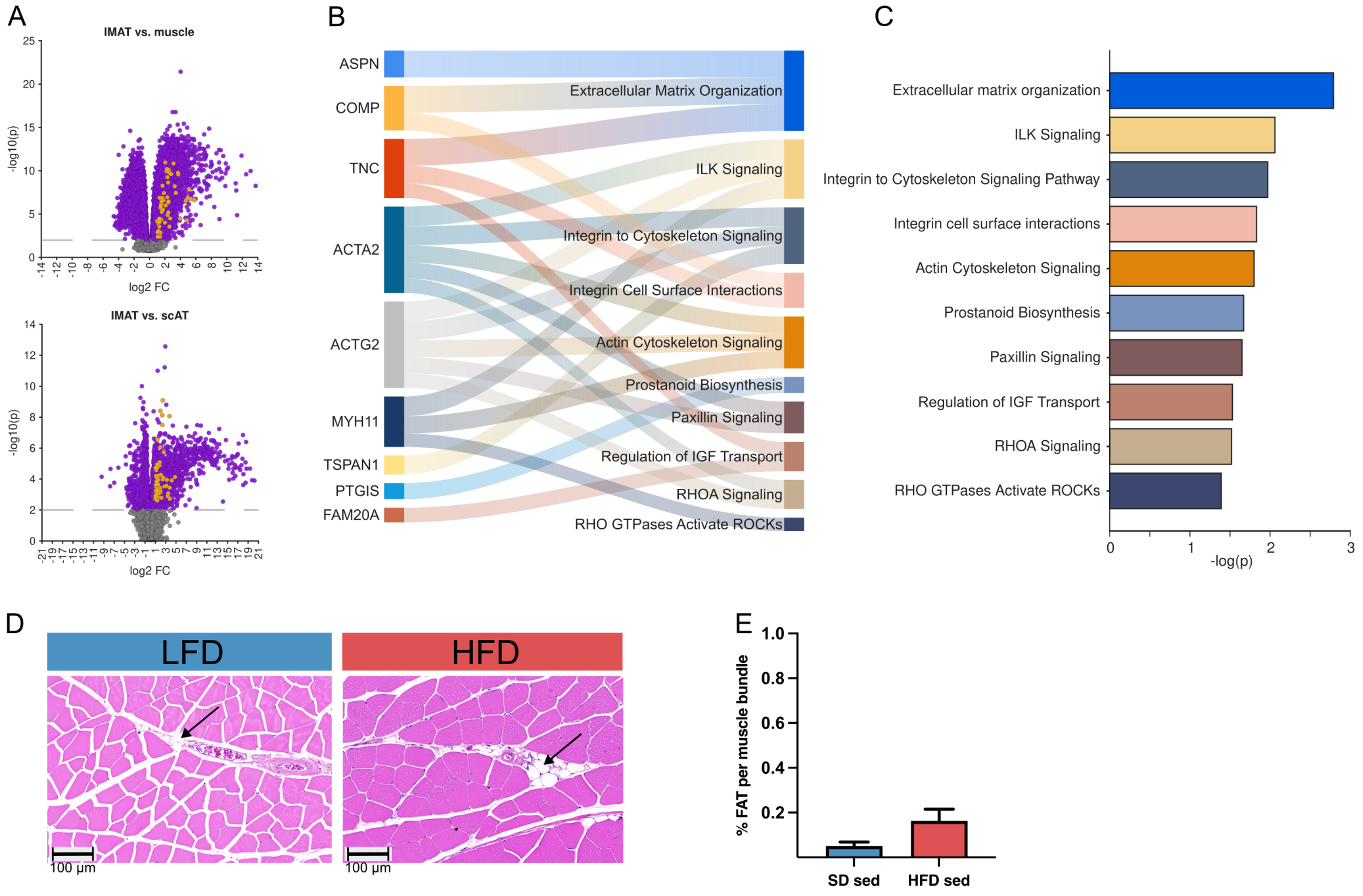

Figure S2

medRxiv preprint doi: https://doi.org/10.64898/2026.04.16.26351017; this version posted June 26, 2026. The copyright holder has placed this preprint (which was not certified by peer review) in the Public Domain. It is no longer restricted by copyright. Anyone can legally share, reuse, remix, or adapt this material for any purpose without crediting the original authors.

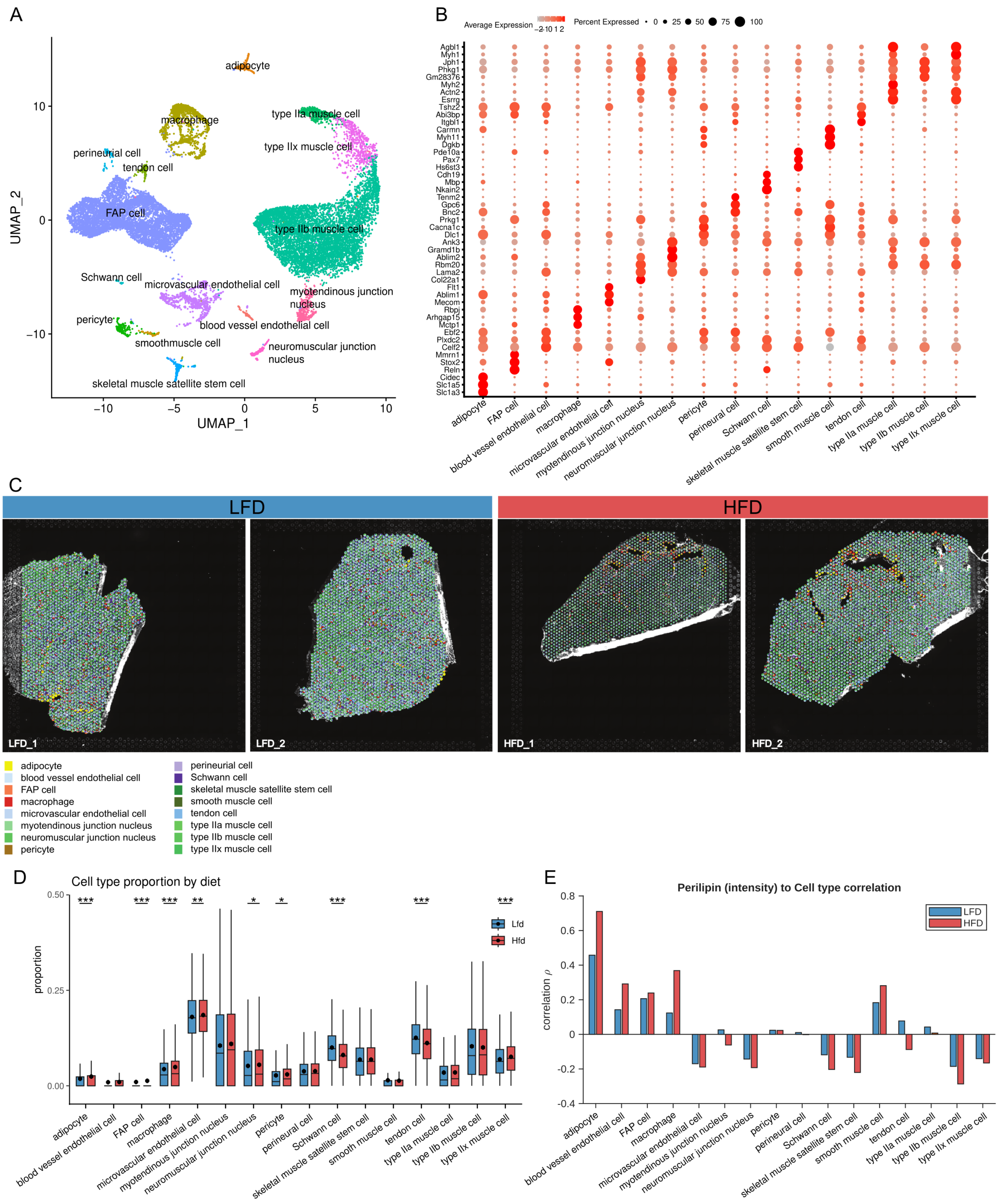

medRxiv preprint doi: https://doi.org/10.64898/2026.04.16.26351017; this version posted June 26, 2026. The copyright holder has placed this preprint (which was not certified by peer review) in the Public Domain. It is no longer restricted by copyright. Anyone can legally share, reuse, remix, or adapt this material for any purpose without crediting the original authors.

Figure S3

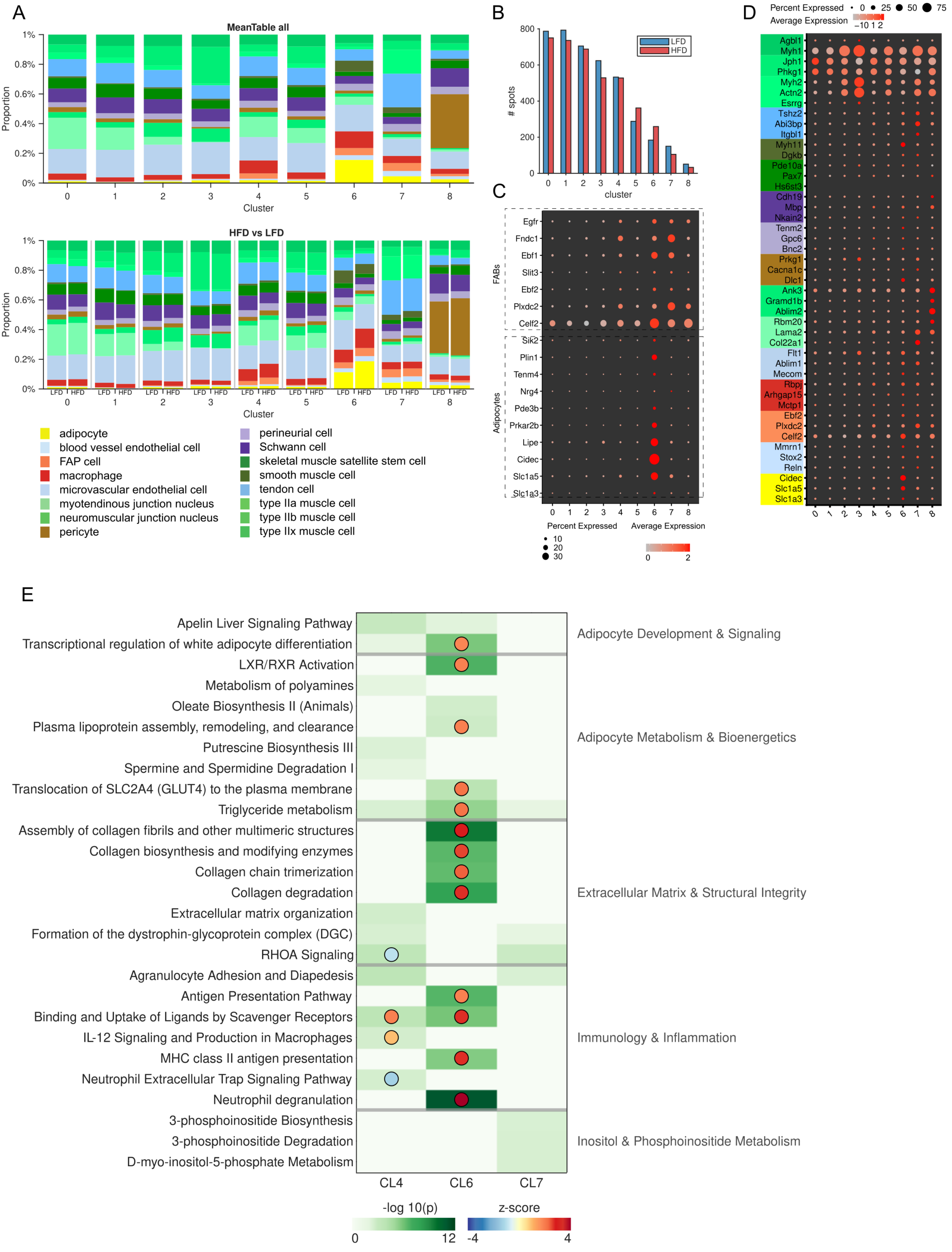

Figure S4

medRxiv preprint doi: https://doi.org/10.64898/2026.04.16.26351017; this version posted June 26, 2026. The copyright holder has placed this preprint (which was not certified by peer review) in the Public Domain. It is no longer restricted by copyright. Anyone can legally share, reuse, remix, or adapt this material for any purpose without crediting the original authors.

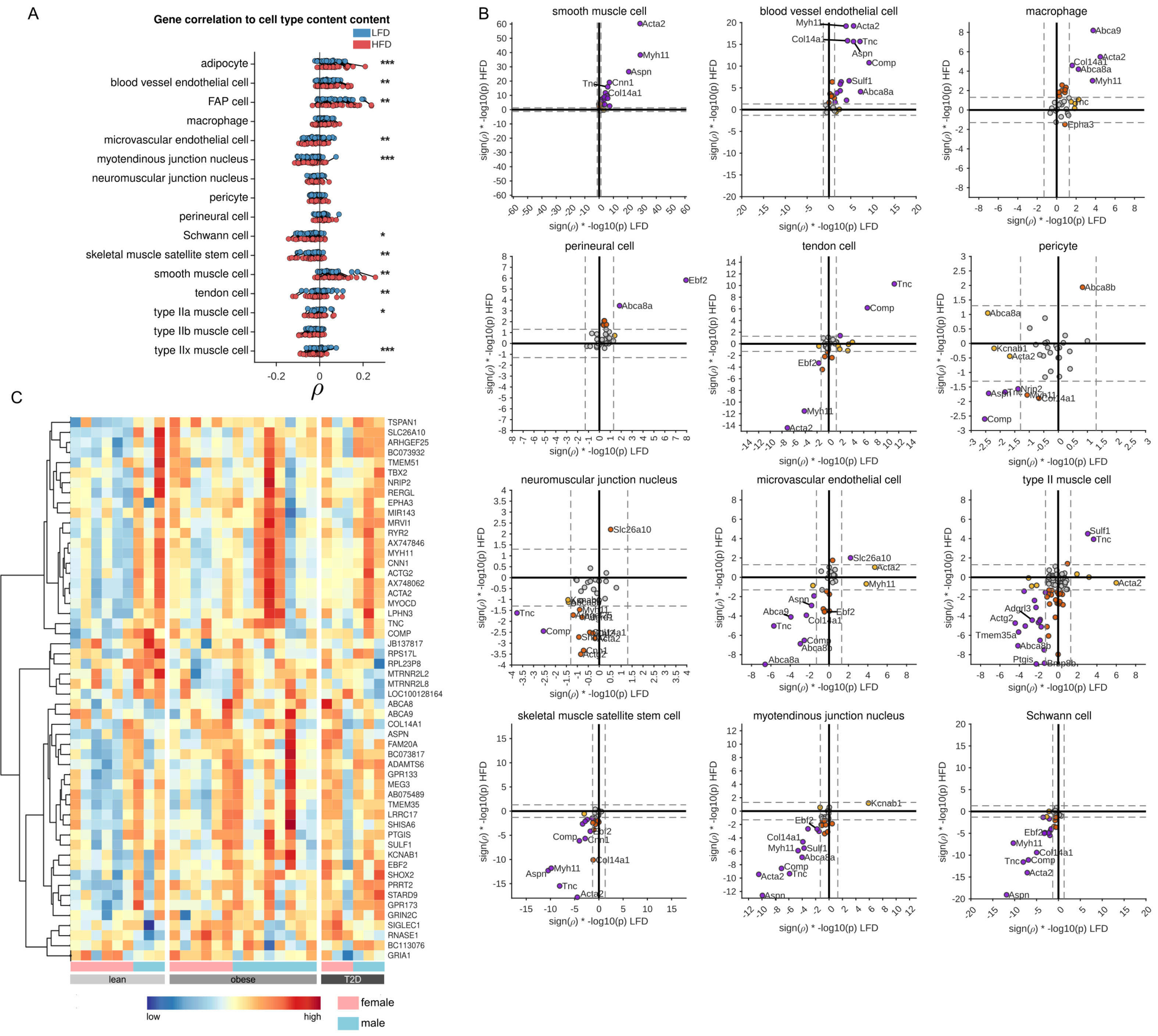

Figure S5

medRxiv preprint doi: https://doi.org/10.64898/2026.04.16.26351017; this version posted June 26, 2026. The copyright holder has placed this preprint (which was not certified by peer review) in the Public Domain. It is no longer restricted by copyright. Anyone can legally share, reuse, remix, or adapt this material for any purpose without crediting the original authors.

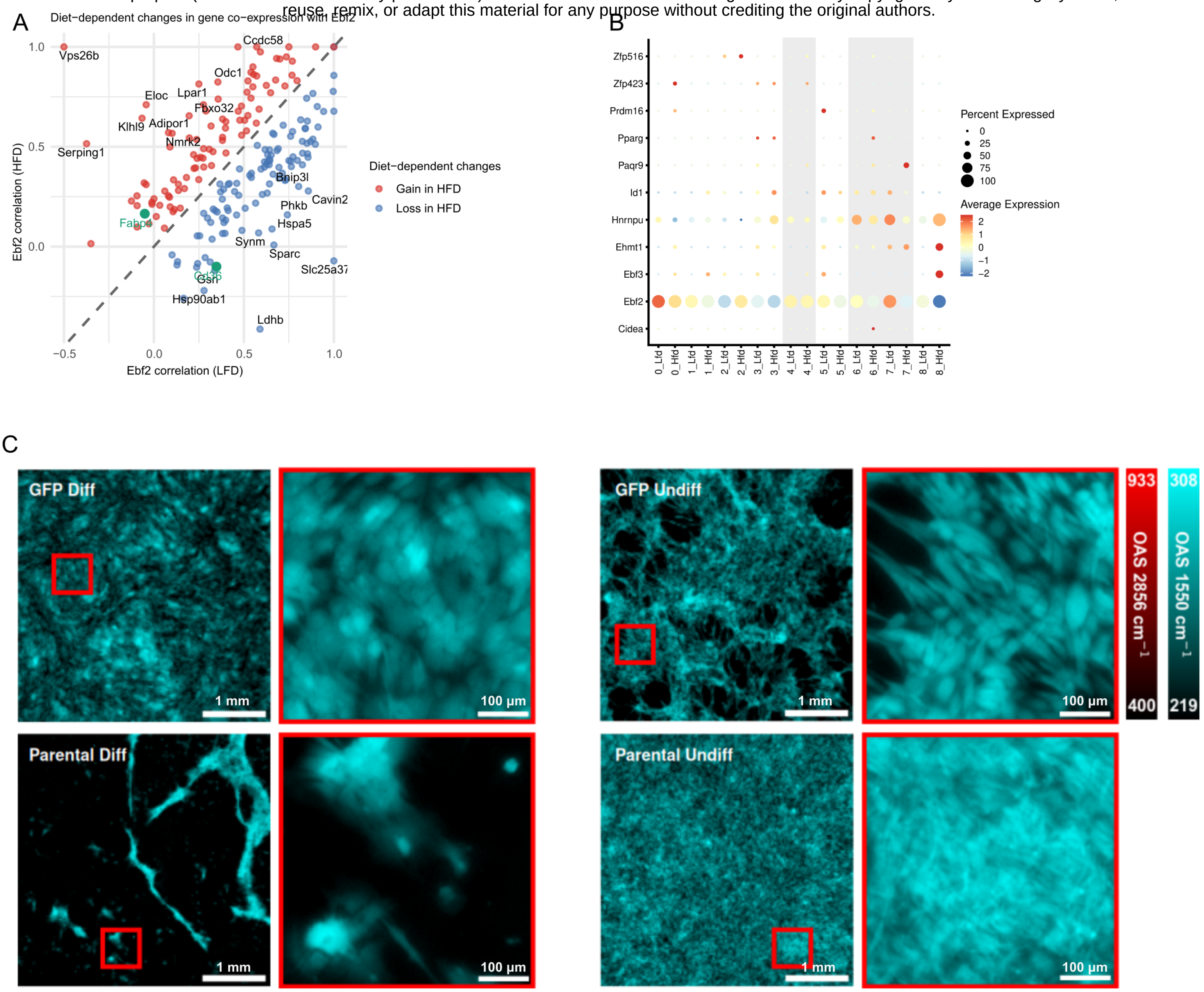

Supplement: Supplement 4 [file NIHPP2026.04.16.26351017v2-supplement-4.pdf]
